# Pharmacology of natural radioprotectors

**DOI:** 10.1007/s12272-018-1083-6

**Published:** 2018-10-25

**Authors:** Gil-Im Mun, Seoyoung Kim, Eun Choi, Cha Soon Kim, Yun-Sil Lee

**Affiliations:** 1 Graduate School of Pharmaceutical Sciences, Ewha Womans University, Seoul, 120-750 Korea; 2 Radiation Health Institute, Korea Hydro & Nuclear Power CO., LTD, 172, Dolmr-ro, Seongnam-si, Gyeonggi-do 13605 Korea

**Keywords:** Natural compounds, Toxicity, Mechanisms, Radioprotectors

## Abstract

Radiotherapy is one of the most efficient ways to treat cancer. However, deleterious effects, such as acute and chronic toxicities that reduce the quality of life, may result. Naturally occurring compounds have been shown to be non-toxic over wide dose ranges and are inexpensive and effective. Additionally, pharmacological strategies have been developed that use radioprotectors to inhibit radiation-induced toxicities. Currently available radioprotectors have several limitations, including toxicity. In this review, we present the mechanisms of proven radioprotectors, ranging from free radical scavenging (the best-known mechanism of radioprotection) to molecular-based radioprotection (e.g., upregulating expression of heat shock proteins). Finally, we discuss naturally occurring compounds with radioprotective properties in the context of these mechanisms.

## Introduction

**Fig. 1:**
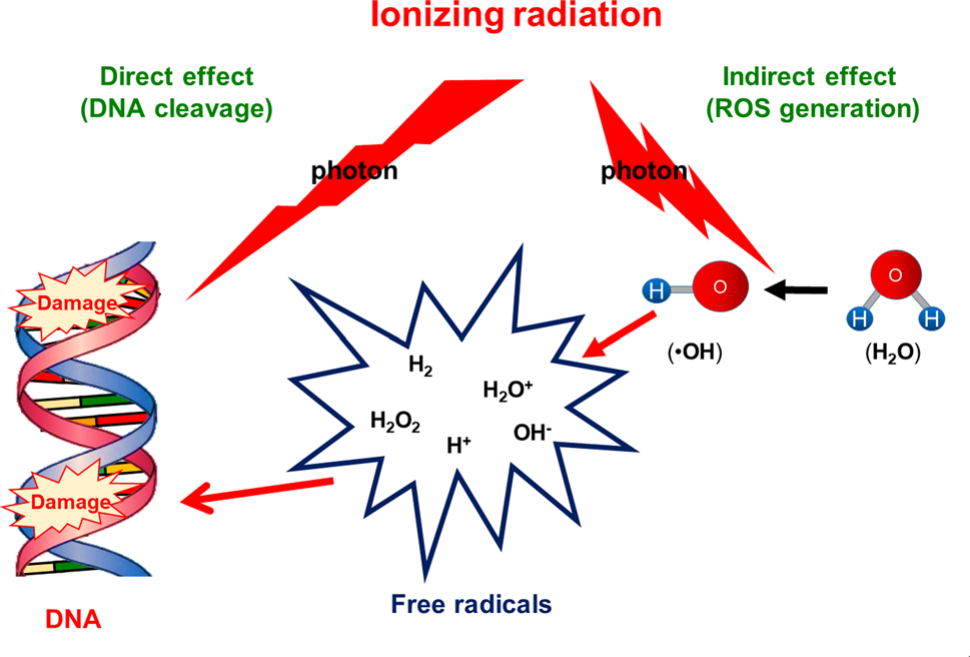
Direct and indirect actions of radiation (modified from Wang et al. 2018). Direct action is mediated by interaction of a secondary electron, resulting from absorption of an X-ray photon, with the DNA. Indirect action is mediated by interaction of a secondary electron with a water molecule to produce reactive oxygen species (ROS), which induces DNA damage

**Fig. 2:**
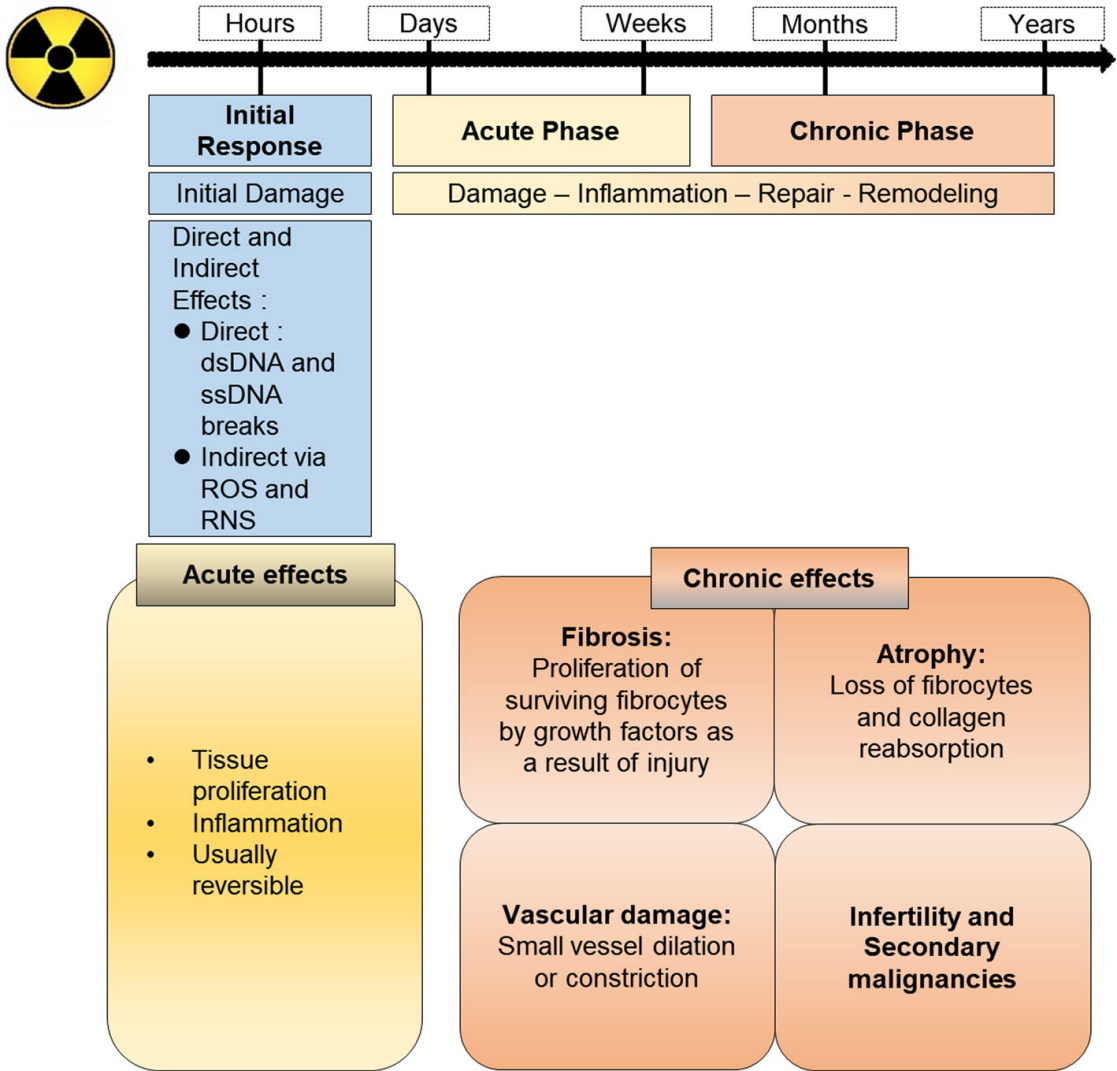
Acute and late effects of radiation exposure (modified from Wirsdorfer and Jendrossek 2016). Radiation effects are commonly divided into acute, or early, and late effects, which are induced after the initial radiation responses. They demonstrate different response patterns to radiation doses. Acute effects result from death of a large number of cells in tissue with rapid rates of turnover and occur within days to weeks of irradiation. Late effects occur months to years after irradiation in tissues with slow cell turnover and usually are persistent and progressive. *dsDNA* double strand DNA, *ssDNA* single strand DNA, *ROS* reactive oxygen species, *RNS* reactive nitrogen species

Radiation therapy is among the most effective treatment modalities for patients with cancer. About 60% of all patients with cancer receive ionizing radiation (IR) as part of their therapeutic regimen (Moding et al. 2013). While IR is a powerful tool for destroying cancer cells, it is also toxic to normal cells and causes cellular damage and unwanted side effects. IR affects biological molecules both directly and indirectly. Direct effects are mediated by direct interaction of IR with individual DNA moieties, and indirect effects occur via reactive oxygen species (ROS) produced from the molecules surrounding DNA (Wang et al. 2018). Because biological systems contain 75-90% water, the indirect effect arises from reaction of water radiolysis products (^·^OH: hydroxyl radicals, solvated electrons, and hydrogen atoms) with DNA. The hydroxyl radical is highly reactive, has powerful oxidizing effects, and can diffuse to react with all cell constituents (Fig. 1). DNA, lipids, and proteins are the main attack targets for hydroxyl radicals. Release of damage-associated molecules and cytokines or chemokines in response to the DNA damage, ROS generation, and apoptosis caused by IR activate the immune system and cause inflammation. This immune activation results in an acute inflammatory phase that is characterized by an enhanced pro-inflammatory response. Inflammation and repair induction after IR are paralleled by mitotic cell death and the subsequent release of cytokines and growth factors, which result in the chronic phase of IR damage (Wirsdorfer and Jendrossek 2016). Many diseases are associated with IR responses, including those caused by acute phase damage (organ inflammation) or chronic phase damage (fibrosis, atrophy, vascular damage, infertility, and secondary malignancies) (Fig. 2).

**Fig. 3:**
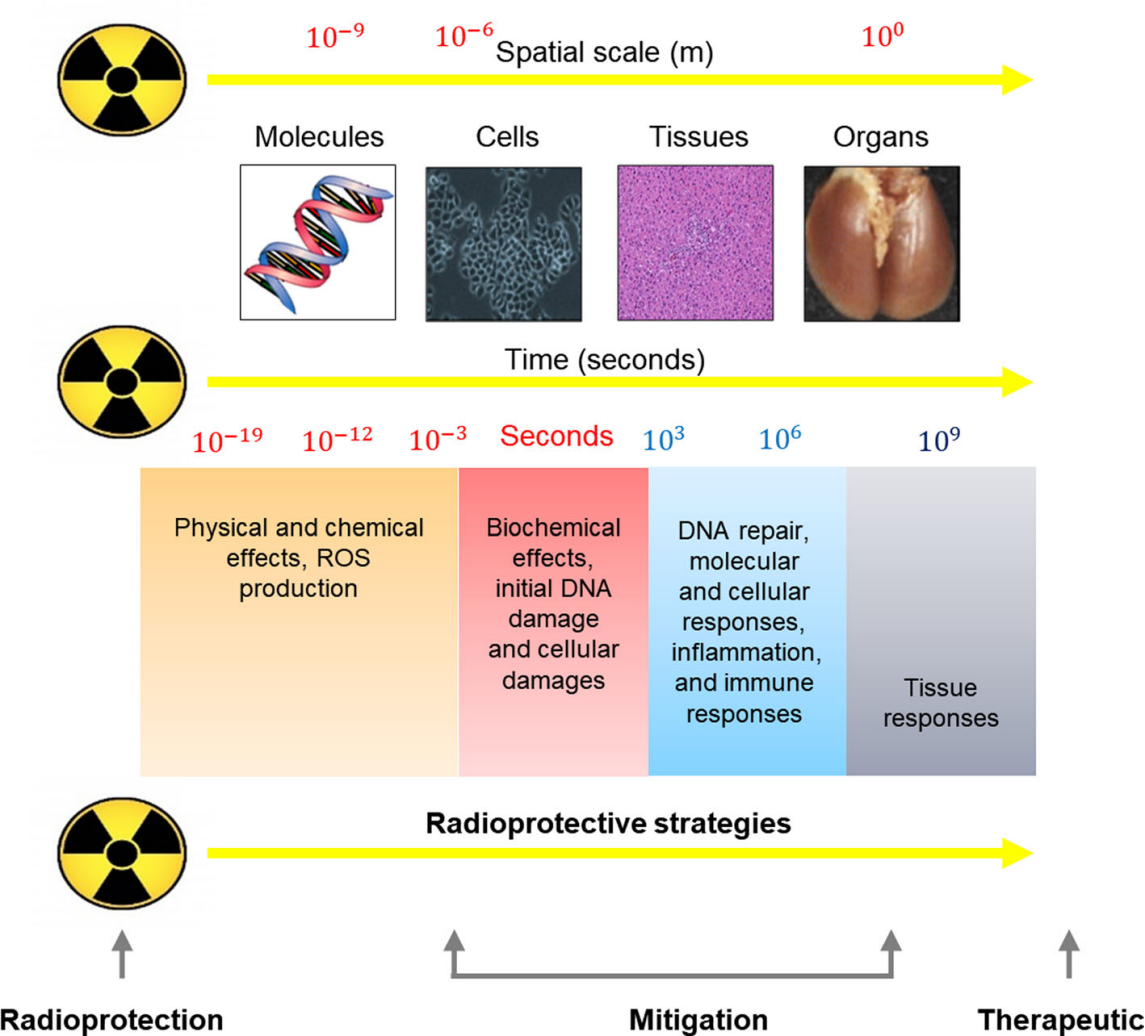
Pharmacological classification of agents with radiation protection properties (modified from Bourgier et al. 2012). Radioprotectors are classified according to when they are administered in relation to radiation and/or its effects. Radioprotectors are administered before radiation exposure to prevent the occurrence of either acute or late effects. Mitigators reduce the action of radiation on normal tissues before radiation emergence syndrome. Therapeutic agents are administered after radiation exposure to treat late effects

There is considerable interest in protecting normal cells from IR. In addition to technological improvements in IR delivery and accuracy, pharmacological agents are being used as an alternative to decrease toxicity to normal tissues. The IR research program of the National Cancer Institute proposed the following pharmacological classification of agents with IR protection properties according to timing of administration: (a) protection, (b) mitigation, and (c) therapeutic agents (Citrin et al. 2010). An ideal radioprotective agent should prevent direct acute or chronic effects on normal tissue, be easily dispensed without toxicity, and not protect tumors from IR. Radioprotectors are used as a prophylactic strategy against chemical effects according to the classification timescale proposed. They are administered before radiotherapy or IR exposure to prevent the occurrence of either acute or chronic effects. Mitigators reduce the IR effects on normal tissues before emergence of symptoms and are administered during or shortly after radiation therapy or IR exposure. Lastly, therapeutic agents are used for treatment following IR to reduce deleterious or chronic effects and are administered after symptoms have presented (Bourgier et al. 2012). Therapeutic agents were initially developed in case of accidental IR exposure and were later adapted to treat acute or chronic effects following IR treatment (Fig. 3). A number of compounds have been studied for potential as radioprotectors, mitigators, and therapeutic agents, and the compounds currently approved by the U.S. Food and Drug Administration (FDA) or in the FDA-Investigational New Drug (IND) application process are described in Table 1.

**Table 1:** FDA approved- or IND application drugs for radioprotectors, mitigators, and therapeutic agents

Categories	Generic name (chemical names and pharmaceutical labels)	Specific use	FDA status	References
Nutraceuticals-Isoflavonoids	Genistein (4′5,7 trihydroxy-isoflavonoid; Bio 300™)	Survival and organ system protection (i.e., marrow, gut, and lung)	IND	Day et al. (2008) Landauer et al. (2003) Zhou and Mi (2005)
Immunomodulator-TLR-5 agonist	CBLB502 (bioengineered truncated Salmonella sp. flagellin; Entolimod™)	Enhancement of survival and protection of marrow and gut damage in selective animal models	IND	Burdelya et al. (2008) Krivokrysenko et al. (2012)
Cortical steroid metabolite	5-AED (androst-5-ene-3β, 17β-diol; Neumune^®^)	Enhancement of survival and protection of bone marrow damage	IND	Stickney et al. (2007) Whitnall et al. (2005)
Corticosteroid	BDP/SGX201 (corticosteroid-beclomethasone 17,21-dipropionate; OrbeShield™)	Mitigation of radiation enteritis and promotion of survival	IND	Singh et al. (2015)
Free-radical quencher-Meso-prophyrin mimetic	AEOL 10150 (Mn prophyrin SOD mimic)	Mitigation of lung damage; upregulates antioxidant system	IND	Garofalo et al. (2014) Orrell (2006)
Recombinant growth factor	rhu G-CSF (filgrastim; Neupogen^®^)	Hemopathological indication; stimulation of neutrophil production; risk reduction of life-threatening infections	IND	Farese et al. (2013) Gourmelon et al. (2010)
Recombinant cytokines	Rhu IL-12 (HemaMax™)	Experimental IND status; enhancement of survival and protection of marrow and gut damage	IND	Basile et al. (2012) Gluzman-Poltorak et al. (2014) Xiong et al. (2013)
Binding agents	Prussian Blue (Radiogardase^®^)	Chelating agent for Cesium-137	Approved	Hussar (2005)
Blocking agent	KI	Thyroid uptake/blocking agent for radio-iodides (Iodine-131, Iodide-125)	Approved	Hammond et al. (2007)
5-hydroxy tryptamine antagonist	Granisetron (Kytril^®^)	Minimizing emetic effects of acute radiation exposure	Approved	Lanciano et al. (2001)
Chemical protectants	Amifostine/WR2721 (2-(3-aminopropyl) aminoethylphosphorothioate; Ethyol™)	Cytoprotection (survival protection; systemic protection of organ systems, but specifically marrow and gut)	Approved	Culy and Spencer (2001) Ormsby et al. (2014)
Cytokines and growth factor	Palifermin (Kepivance^®^)	Stimulation of differentiation, proliferation, DNA repair, and detoxification of ROS	Approved	Finch and Rubin (2004)

*TLR* Toll-like receptor, *AED* androstenediol, *SOD* superoxide dismutase, *BDP* beclomethasone 17,21-dipropionate, *G*-*CSF* granulocyte colony stimulating factor

**Fig. 4:**
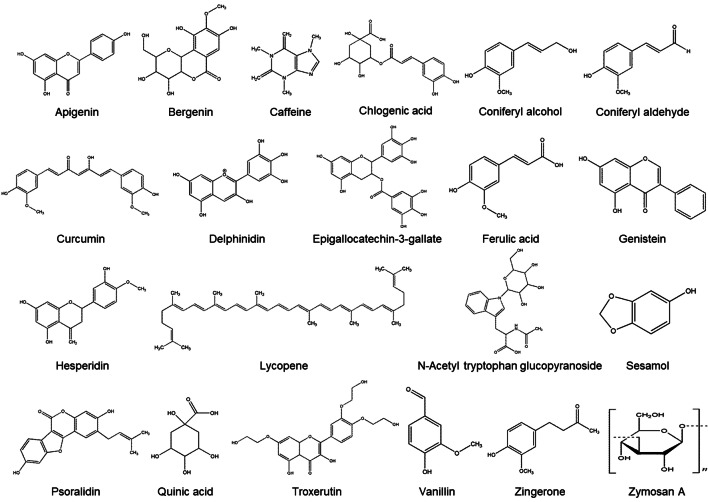
Chemical structures of naturally occurring compounds with radioprotective effects

**Fig. 5:**
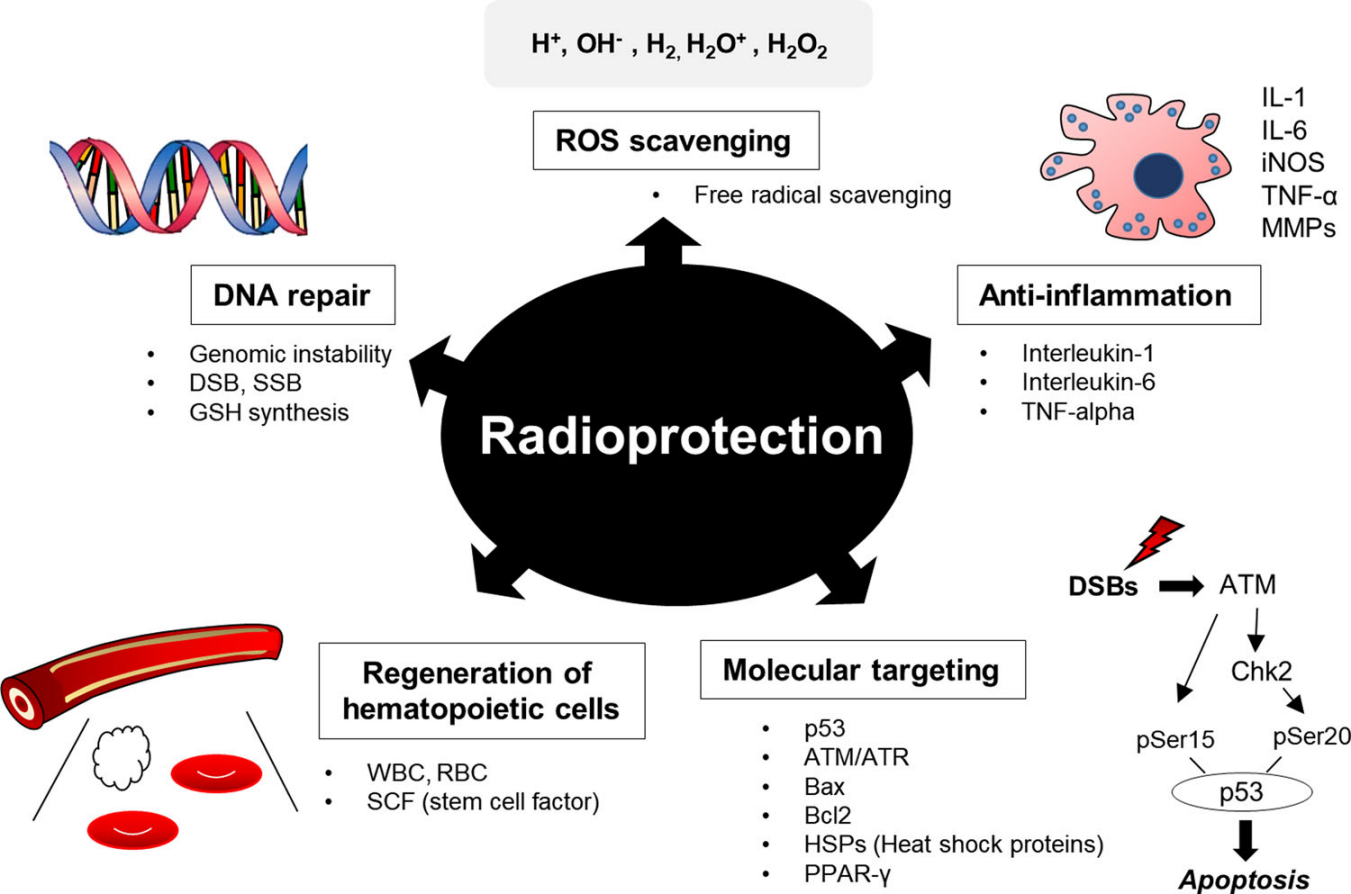
Summary of the cellular mechanisms of naturally occurring compounds with radioprotective effects. Naturally occurring compounds with radioprotective effects occur through different mechanisms, such as free radical scavenging, anti-inflammation, facilitation of repair activity, regeneration of hematopoietic cells, and affecting to molecular levels. The mechanisms of radioprotectors are involved in radiation response and tissue damage repair, which may be potential pharmacological targets for development of ideal radioprotectors. *DSB* double strand break, *SSB* single strand break, *WBC* white blood cells, *RBC* red blood cells

Over the last few decades, many natural and synthetic compounds have been investigated for their potential as radioprotectors. However, amifostine, developed by the U.S. Army Anti-Radiation Drug Development Program, is the only chemical radioprotector currently approved by the FDA. Amifostine has disadvantages such as limited administration routes, narrow administration window for efficacy, high cost, and limited protection of organs (Cheki et al. 2016). Thus, while chemical radioprotectors remain a subject of active research, their efficacy is often limited by high toxicity, side effects, and high cost. Due to the inherent toxicity of synthetic chemicals, an interest in natural plants and phytochemicals as a potential source of radioprotectors has developed. Naturally occurring compounds with discrete bio-activities are being widely examined for their potential ability to provide health benefits, and a number of naturally occurring compounds have demonstrated radioprotective activities. Since naturally occurring compounds are typically less toxic than synthetic compounds and can be less expensive, they are preferable sources of radioprotectors. Therefore, screening of naturally occurring compounds is a major focus for new drug discovery. This review evaluates the radioprotective effects of naturally occurring compounds and the mechanisms underlying these effects (Table 2; Figs. 4, 5).

**Table 2:** List of naturally occurring compounds with radioprotective effects

Natural compounds	Source	Radioprotective effects	References
Apigenin	Parsley, Celery, Chamomile	Anti-inflammatory, anti-proliferative, and anti-progression	Begum et al. (2012)
Bergenin	*Caesalpinia digyna*	Activation of the MAP kinase and ERK pathways and protection against radiation damage	Dwivedi et al. (2017) Veerapur et al. (2009)
Caffeine	Coffee beans	Protection against the oxic component of damage in rat liver mitochondria Inhibition of radiation-mediated chromosomal aberrations in mouse bone marrow cells	Hall and Giaccia (2012)
Chlorogenic acid/quinic acid	Cinchona bark, Coffee beans	Anti-inflammation, anti-mutagenic, DNA damage inhibition, and anti-oxidation	Cinkilic et al. (2013)
Coniferyl aldehyde	*Eucommia ulmoides*	Induction of heat shock transcription factor 1 and protection against radiation damage	Kim et al. (2015) Nam et al. (2013)
Curcumin	Turmeric root	Reduction of gastrointestinal symptoms during chemotherapy and radiation therapy Reduction of mucositis during radiation therapy Reduction of radiation dermatitis and desquamation	Verma (2016)
Delphinidin	Carrot, Tomato, Red onion, Cranberries, Concord grapes, etc	Anti-oxidation and anti-inflammation	Watson and Schonlau (2015) Jeong et al. (2016)
Epigallocatechin-3-gallate	*Camellia sinensis*	Increased levels of several anti-oxidant enzymes Protection of skin cells against radiation-induced damage and radioprotective effects against several radiation-mediated responses	Zhu et al. (2016) Zhang et al. (2016)
Ferulic acid	Rice, Green tea, Coffee beans	Protection against radiation-induced damage and enhancement of DNA repair Prevention of radiation-induced micronuclei and dicentric aberrations in human lymphocytes Enhancement of survival in mice after radiation	Das et al. (2014) Kikuzaki et al. (2002) Zhao et al. (2003)
Genistein	*Genista tictoria*, etc.	Protection against acute radiation injury Multiple mechanisms (e.g., antioxidant, free radical scavenger, anti-inflammatory, activation of the DNA repair enzyme Gadd45	Davis et al. (2007) Grace et al. (2007)\ Ahmad et al. (2010)
Hesperidin	Citrus fruit	Efficient radioprotection in rat lung tissue Protection of lipid peroxidation during radiation-induced tissue damage in rats	Fardid et al. (2016) Rezaeyan et al. (2016)
Lycopene	Tomato, Watermelon, Pink grapefruit, Papaya, etc	Protection against radiation-induced chromosomal damage in human lymphocytes Increased survival after radiation exposure	Kelkel et al. (2011) Srinivasan et al. (2009)
N-Acetyl tryptophan glucopyranoside (NATG)	*Bacillus subtilis*	Overcoming radiation-induced apoptosis by improving cytoprotective cytokines Enhancement of antioxidant enzymes against radiation-induced damage	Malhotra et al. (2015) Malhotra et al. (2018)
Psoralidin	*Psoralea corylifolia*	Inhibition of radiation-induced PI3 K-IKK-IκB signaling pathway and COX-2 expression Suppression of radiation-induced expression of pro-inflammatory cytokines	Chiou et al. (2011) Yang et al. (2011)
Sesamol	Sesame seeds, Sesame oil	Strong ROS scavenging and antioxidant properties	Kanimozhi and Prasad (2009) Mishra et al. (2011)
Troxerutin	*Sophora japonica*	Protection against radiation-induced damage to the salivary glands and mucosa Inhibition of lipid peroxidation in the membranes of subcellular organelles Differential protection of normal cells in irradiated tumor-bearing mice: protection in blood leukocytes and bone marrow cells but not in tumor cells	Maurya et al. (2004)
Vanillin	*Vanilla orchid* (*Vanilla planifolia, V. fragrans*)	Suppression of radiation-induced chromosomal aberrations in cells and in mice Anti-mutagenic effects	Kumar et al. (2000)
Zingerone	Ginger	Anti-oxidation	Ahmad et al. (2015) Rao and Rao (2010)
Zymosan A	*Saccharomyces cerevisiae*	Protection from radiation-induced apoptosis by upregulating the levels of cytokines Protection of cells from radiation-induced DNA damage	Du et al. (2018)

## Underlying mechanisms of radioprotectors

### Antioxidation

During radiation exposure, IR generates free radicals and ROS, which result in cellular damage. Free radicals induce DNA damage by introducing base damage, DNA double-strand breaks (DSBs), and DNA–DNA or DNA–protein cross-links. These effects can alter gene expression and cause protein modifications, cell death, senescence, and genomic instability. Exposure of cells to a typical clinical dose of IR has been shown to result in an average of 1000 DNA single-strand breaks (SSB), 40 DNA DSBs, and 3000 damaged bases per Gray (Gy) (Hall and Giaccia 2012). Since most of this IR damage arises from interaction of IR-induced free radicals with biomolecules, agents that can destroy free radicals or prevent formation of free radicals can inhibit these reactions and function as radioprotectors. Free radicals are short-lived and interact rapidly with biomolecules (Lobo et al. 2010). Therefore, to provide proper radioprotection, effective molecules need to be present in the cellular systems at a sufficient concentration at the time of IR exposure.

### Anti-inflammation

IR is indirectly toxic by activating an immune response, and patients undergoing radiation therapy often suffer from widespread inflammation. To improve patient compliance, it is important to relieve the inflammation-associated side effects, and some natural products and their active ingredients can achieve this through anti-inflammatory activity. After exposure to IR, various pro-inflammatory cytokines and chemokines such as interleukin-1, interleukin-6, tumor necrosis factor α, and transforming growth factor β (TGF-β) are generated (Di Maggio et al. 2015). TGF-β is of particular importance in IR damage because this cytokine mediates IR-induced fibrosis of the lungs and skin (Straub et al. 2015). For example, mice fed flaxseed (which is found in *Linum usitatissimum*) had reduced expression of TGF-β and lung injury biomarkers such as Bax and p21, and this was accompanied by reduced lung inflammation and lung fibrosis after radiation therapy (Lee et al. 2009).

### DNA repair and cell recovery processes

Oxidative free radicals can damage DNA by introducing SSB, DSB, and base lesions. While it is important to prevent cellular damage, cellular recovery pathways and repair processes can also mitigate this damage. Several studies have suggested that cellular recovery and repair processes can be enhanced by radioprotectors. Specifically, the DNA SSB repair system is absent in cells deficient in glutathione (GSH) synthesis, suggesting that thiols such as GSH might be involved in the repair of DNA SSB. DNA precursor-synthesizing enzymes such as ribonucleotide reductase also have an important role in DNA repair, and mammalian ribonucleotide reductase activity is induced by DNA damage. Specifically, this enzyme maintains the deoxyribonucleotide concentration, which is important in the excision repair process (Sharygin et al. 2005; Thelander 2007). Overall, higher cellular pools of DNA precursors can create a radioprotective cellular environment, and drugs and chemicals that stimulate the activity of precursor-synthesizing enzymes can function as radioprotectors.

### Regeneration (hematopoietic and immunostimulant compounds)

IR exposure leads to dose-dependent defects of the lymphoid and hematopoietic systems through a complex cascade known as hematopoietic syndrome, which can result in septicemia and death (Guo et al. 2015). Hence, modulating the regeneration of hematopoietic cells and stimulating the immune system (e.g., by increasing the number of spleen colony-forming units) are effective therapeutic strategies for overcoming IR-induced damage. As such, various endogenous compounds such as immune-modulators, growth factors, and cytokines have been found to be effective radioprotectors. For example, the endogenous agents IL-1, TNF-α, granulocyte colony-stimulating factor (G-CSF), stem cell factor (SCF), erythropoietin (EPO), and granulocyte-macrophage colony-stimulating factor (GM)-CSF have been investigated as potential radioprotectors. These agents stimulate stem cell progenitors and promote hematopoietic bone marrow (BM) repopulation (Dumont et al. 2010; Schaue et al. 2012). Hence, agents that upregulate endogenous radioprotective factors can also act as radioprotectors.

### Molecular-based radioprotection

Recent studies have focused on understanding the signaling and apoptotic pathways involved in IR damage and identifying the events that occur late in these pathways as potential targets for post-irradiation intervention. For instance, ATM/ATR, a sensing protein and p53 tumor suppressor, regulates the DNA damage pathway. ATM/ATR plays a key role in triggering apoptotic cell death by upregulating pro-apoptotic proteins such as Apaf-1, Noxa, and Bax after IR. Recent studies demonstrated that pifithrin-μ, a small-molecule inhibitor of p53, protected thymocytes from IR-induced apoptosis and reduced IR-induced death when injected into mice before IR exposure (Strom et al. 2006). Similarly, overexpression of Bcl2, an inhibitor of pro-apoptotic proteins, in transgenic mice has also been shown to effectively increase the survival of irradiated mice by protecting against IR-induced apoptosis in hematopoietic cells (Erlacher et al. 2005). In addition, some studies have suggested that STAT3 protects against IR damage, and STAT3 can be activated by various growth factors. For example, the STAT3 activator CBLB502 was recently demonstrated to protect mice from IR-induced damage, and another study showed that STAT3 activation protects hair cells from IR-induced cell death (Xu et al. 2016). Nuclear factor-erythroid 2-related factor 2 (Nrf2) is a key transcriptional regulator of antioxidant and anti-inflammatory enzymes. Genetic upregulation of Nrf2 in the skin of SKH-1 hairless mice has been shown to protect against IR damage (Knatko et al. 2015). Importantly, heat shock proteins (HSPs) are cytoprotective and can mediate cell and tissue repair after deleterious effects of IR (Lee et al. 2001). Overexpression of one or more HSP genes has been shown to be sufficient to protect against IR exposure, and small molecules that can enhance the expression or function of HSPs have also shown promise for treatment of chronic or acute symptoms after IR. The radioprotective effects of HSPs have been shown to be related to apoptotic pathway interference (Akerfelt et al. 2010; Kim et al. 2006). Peroxisome proliferator-activated receptor-γ (PPAR-γ) is part of the nuclear hormone receptor family, and PPAR-γ agonists inhibit collagen deposition and TGF-β1-induced collagen secretion in bleomycin-induced pulmonary fibrosis (Milam et al. 2008). Rosiglitazone, a PPAR-γ synthetic activator, suppresses IR-induced survival signals and DNA damage responses and enhances IR-induced apoptosis signaling in human cells (Mangoni et al. 2017). Moreover, PPAR-γ is suggested to be an important regulator of fibroblast/myofibroblast activation. Therefore, PPAR-γ ligands have also been suggested as novel therapeutic agents for IR-induced fibrotic lung diseases.

## Naturally occurring radioprotectors

Amifostine is currently the only chemical drug approved by the FDA for protecting against the toxicity of radiotherapy in cancer patients. The free radical scavenger amifostine is an organic thiol phosphate prodrug and a chemical radioprotector similar to thiols, aminothiols, thiadiazoles, and benzothiazoles. However, the efficacy of such chemical radioprotectors is limited by their high toxicity and associated side effects (Andreassen et al. 2003). Indeed, amifostine has several clinically relevant limitations, including (1) an administration time within a narrow window (15–30 min before IR exposure); (2) approval only for intravenous (IV) administration (Yu et al. 2003); and (3) high toxicity associated with undesirable side effects including nausea, vomiting, cephalalgia, and hypotension.

However, many natural compounds, such as hormones and vitamins, also confer some level of radioprotection. For example, β-glucagon and the polysaccharide ginsan have multiple immunomodulatory effects and radioprotective activity (Luo and Luo 2009). Moreover, 5-androstenediol, a hormone produced by the adrenal cortex, and vitamins C and E have also been shown to exhibit radioprotective properties (Gonzalez et al. 2018; Tabeie et al. 2017; Whitnall et al. 2001). Some natural compounds including vitamin C, glutamine, arginine, ubiquinone, and hydroquinone have been investigated for their ability to protect the immune system against IR. However, further in vivo and in vitro studies are necessary to validate these compounds as effective natural radioprotectors (Painuli and Kumar 2016).

Therefore, focus has shifted to the evaluation of natural product-based radioprotectors, given that they are less toxic, highly efficient, and inexpensive. For example, polyphenols, flavonoids, and a range of secondary metabolites are found in different plant parts and have radioprotective properties (Citrin et al. 2010; Pal et al. 2013). This review evaluates the radioprotective effects of several plant-derived compounds based on studies from the last 15–17 years that assessed their radioprotective potential and mechanisms.

### Apigenin

Apigenin (4′,5,7-trihydoxyflavone) is one of the most common flavonoids and is widely distributed throughout the leaves and stems of many dietary vegetables and fruits, including bell peppers, broccoli, celery, Chinese cabbage, French peas, garlic, guava, leeks, onions, tomatoes, snake ground, and wolfberry leaves (Miean and Mohamed 2001). It is also found in plant-derived beverages such as tea and wine (Shukla and Gupta 2010). Apigenin significantly decreases the frequency of IR-induced micronuclei (Rithidech et al. 2005). Additionally, apigenin treatment before IR significantly reduced DNA damage and nuclear buds in irradiated human peripheral blood lymphocytes, suggesting that apigenin protects lymphocytes from IR-induced cytogenetic alteration (Begum et al. 2012).

### Bergenin

Bergenin has been isolated from the root extract of *Caesalpinia digyna*. This compound activates various signaling pathways, such as the ERK1/2, MAP kinase, and SAPK/JNK pathways, and also induces TNF-α, nitric oxide (NO), and IL-12 production in infected murine macrophages. Bergenin is a hydrolysable tannin derivative and exhibits anti-hepatotoxic, anti-ulcerogenic, anti-HIV, anti-arrhythmic, anti-malarial, anti-inflammatory, neuroprotective, and immunomodulatory effects (Dwivedi et al. 2017). Bergenin effectively protects against DNA damage, and it has been suggested that its hydroxyl radical scavenging activity is critical for this DNA damage protection (Veerapur et al. 2009).

### Caffeine

Caffeine, a methyl xanthine derivative, has also demonstrated protective activities against IR damage (Kim et al. 2003). A cohort study of patients receiving radiotherapy for cervical cancer found an inverse correlation between caffeine ingestion at the time of radiation therapy and incidence of severe IR toxicities (Stelzer et al. 1994). Animal studies have also evaluated the effects of caffeine on mouse survival following exposure to lethal doses of IR. Caffeine decreased IR-induced skin damage associated with radiation therapy (Hebbar et al. 2002) and conferred radioprotection against IR-induced damage in hamster ovary cells (Kesavan and Natarajan 1985), rat liver mitochondria (Kamat et al. 2000), and plasmid DNA (Kumar et al. 2001). It has also been shown to have radioprotective properties in the BM chromosomes of mice, regardless of whether it was given before or after whole body IR (Farooqi and Kesavan 1992). Caffeine has both antioxidant and anti-inflammatory properties (Hall et al. 2015), which may contribute to its protective effects against IR-induced damage. Specifically, caffeine scavenges hydroxyl radicals (Brezova et al. 2009) and competes with oxygen for IR-induced electrons. Moreover, caffeine restores the normal cell cycle following IR-induced arrest in the G2 phase of mouse embryos (Grinfeld and Jacquet 1987), and this effect was dependent on protein synthesis (Jung and Streffer 1992). Finally, caffeine was demonstrated to reduce the amount of UV radiation-induced protein in melanoma cells (Ravi et al. 2008).

### Chlorogenic acid and quinic acid

Chlorogenic acid (5-*O*-caffeoylquinic acid) is the ester form of caffeic acid and quinic acid and is in the hydroxycinnamic acid group (Chandrasekara and Shahidi 2010; Santana-Galvez et al. 2017). Chlorogenic acid is an important plant polyphenol and is widely distributed in the leaves and fruit of coffee beans. It is hydrolyzed by internal microflora into various aromatic acid metabolites including caffeic acid and quinic acid (Gonthier et al. 2003). Quinic acid (1,3,4,5-tetrahydroxycyclohexane carboxylic acid) is a naturally occurring polyphenol distributed in cocoa beans, coffee, wine, and fruits and can be chemically synthesized from chlorogenic acid. Coffee beans are the main source of chlorogenic acid and quinic acid (Santana-Galvez et al. 2017). In one study, the alkaline comet assay demonstrated that quinic acid and chlorogenic acid protected against DNA damage induced by IR, suggesting significant radioprotective effects of these compounds (Cinkilic et al. 2013).

### Coniferyl aldehyde and coniferyl alcohol

Coniferyl aldehyde (CA) is isolated from the bark of *Eucommia ulmoides* Oliver and has been shown to induce heat shock transcription factor 1 (HSF1) and protect against IR-induced damage. CA also increases the stability of HSF1 by phosphorylating it at residue Ser326, resulting in increased expression of HSP27 and HSP70. Moreover, EKR1/2 activation was demonstrated to mediate HSF1 phosphorylation at Ser326 by CA. Simultaneous exposure of normal cells to CA and IR or the chemotherapy medication paclitaxel demonstrated protective effects of CA that were dependent on HSF1 Ser326 phosphorylation. Furthermore, IR-mediated decrease in BM cellularity and IR-mediated increase in terminal deoxynucleotidyl transferase dUTP nick end labeling (TUNEL)-positive BM cells were both significantly inhibited by CA in mice. Studies using an A549 orthotopic lung tumor model indicated that CA did not affect IR-mediated decrease in lung tumor nodules while normal lung tissue was protected from irradiation. Taken together, these studies suggest that CA could be used to induce HSF1 and protect normal cells against IR- and/or chemotherapeutic agent-induced damage (Kim et al. 2015; Nam et al. 2013). Another study demonstrated that coniferyl alcohol (COH), a hydroxyl derivative of CA, also activated HSF1. Further investigation demonstrated that the vinyl moiety of CA and COH is the pharmacophore essential for inducing HSF1, which may be useful in future studies for developing small molecules for cytoprotection of normal cells against IR damage (Choi et al. 2017).

### Curcumin

Curcumin is mainly extracted from the roots of *Curcuma lona* and has been demonstrated to exert radioprotective effects and to possess antitumor properties (Perrone et al. 2015). For example, curcumin reduced pulmonary fibrosis and increased the survival of mice exposed to IR. Curcumin acts as a free radical scavenger, inhibiting IR-induced ROS generation (Verma 2016). Another study demonstrated that IR-induced cell death was decreased by curcumin treatment in MCF7 breast cancer cells (Swati et al. 2009). Curcumin has also demonstrated the dual actions of radioprotecting non-cancerous normal cells while radiosensitizing tumor cells, which was found to be mediated by its radical scavenging properties and cell cycle checkpoints (Sebastia et al. 2014). Additionally, curcumin ameliorated IR-induced cognitive deficits, including learning and memory defects, by enhancing the Nrf2 antioxidant signaling pathways (Xie et al. 2014). Recent study has also demonstrated that curcumin-based therapeutic nanoparticles protect normal cells by scavenging free radicals (Xie et al. 2017). Finally, there is evidence that curcumin is a potent anti-inflammatory molecule (Menon and Sudheer 2007; Okunieff et al. 2006) and increases the balance of antioxidants to oxidants (Tawfik et al. 2013).

### Delphinidin

Delphinidin is an anthocyanidin compound that possesses strong anti-inflammatory and antioxidant effects, among other various biological activities (Jeong et al. 2016). It is abundantly found in pigmented vegetables, such as carrots, tomatoes, and red onions, and fruits, including cranberries and concord grapes (Yun et al. 2009). Among the anthocyanins, delphinidin demonstrates the strongest antioxidant activity due to the many hydroxyl radicals in its structure (Watson and Schonlau 2015). Delphinidin also protects normal tissues from high linear energy transfer radiation such as that of protons (Kim et al. 2018). Taken together, these properties suggest that delphinidin is a promising radioprotector.

### Epigallocatechin-3-gallate

Epigallocatechin-3-gallate (EGCG) is the main polyphenolic constituent of green tea and is widely recognized as a powerful free radical scavenger. Studies have shown EGCG to be effective in treating several disorders (Zhu et al. 2016). Specifically, EGCG has demonstrated anti-aging, anti-angiogenic, anti-arthritic, antiviral, anti-inflammatory, and neuroprotective effects. It has also been reported that EGCG increases the levels of several antioxidant enzymes, including glutamate cysteine ligase, superoxide dismutase (SOD), and heme oxygenase-1 (HO-1) (Zhu et al. 2014), both in vitro and in vivo. Moreover, EGCG can decrease the radiosensitivity of cell systems and enhance DNA repair activity following sublethal IR damage (Pianetti et al. 2002). Furthermore, pretreatment with EGCG was shown to significantly enhance cell viability, maintain cell mitochondrial mass, and decrease the levels of apoptosis and ROS induced by IR in keratinocytes. The protective effects of EGCG in response to UV radiation, which consequently inhibit cutaneous photoaging, have been widely reported. In addition, one mouse study reported that EGCG demonstrated radioprotective effects against IR-induced damage as measured by the spleen index, hematological parameters, malondialdehyde level, and SOD activity (Guvvala et al. 2017; Zhang et al. 2016).

### Ferulic acid

Ferulic acid is a monophenolic phenylpropanoid that occurs naturally in plants such as coffee beans, green tea leaves, and rice. Ferulic acid possesses anti-inflammatory and antioxidant activity that indicate potential protective effects against IR-induced toxicity (Das et al. 2014). Indeed, in vitro studies have demonstrated that the antioxidant properties of ferulic acid protect against IR-induced toxicity. Specifically, ferulic acid shows antioxidant activity against peroxyl radical-induced oxidation in neuronal culture and synaptosomal membranes (Kanski et al. 2002). Ferulic acid also scavenges ROS, such as hydroxyl radicals (^∙^OH) and peroxyl radicals (RO_2_^∙^), as well as the stable free radical 1,1-diphenyl-2-picrylhydrazyl (DPPH) (Kanski et al. 2002; Kikuzaki et al. 2002). Pretreatment of lymphocytes and hepatocytes with ferulic acid resulted in a significant decrease in DNA damage and lipid peroxidation after IR exposure (Srinivasan et al. 2006). Furthermore, ferulic acid pretreatment significantly enhanced antioxidant defenses by upregulating antioxidant enzymes and reducing GSH level. Similarly, administration of ferulic acid prior to IR was shown to significantly reduce DNA damage in mouse blood leukocytes and bone marrow cells. In one study, ferulic acid given prior to or immediately after IR was shown to significantly reduce the level of micronucleated reticulocytes in mouse blood and to enhance DNA repair in mouse peripheral blood leukocytes (Maurya and Devasagayam 2013). Finally, one short-term feeding study found that ferulic acid-sulfoglucuronide was the main metabolite in plasma when ferulic acid or its sugar esters were administered to rats (Zhao et al. 2003).

### Genistein

Genistein (4′,5,7-trihydroxyisoflavone), a soy isoflavone, has demonstrated protection against IR toxicities. The protective effects of genistein are mediated by a combination of activities within the cell, including free radical-scavenging activity, antioxidant activity, and anti-inflammatory activity (Davis et al. 2007). Genistein demonstrated protection against IR-induced damage to mouse BM by preserving neutrophils and platelets (Zhou and Mi 2005) and also protected BM progenitor cell populations to prevent hematopoietic stem cell pool exhaustion (Davis et al. 2008). Moreover, genistein has been shown to reduce IR damage in the lung and increase survival in mice receiving thoracic radiation (Day et al. 2008). Genistein also decreased the number of micronuclei in BM and lung fibroblasts, suggesting a reduction in IR-induced DNA damage (Mahmood et al. 2011; Para et al. 2009). In addition to the protective activities mentioned above, several other mechanisms have been proposed to mediate the radioprotective effects of genistein, including activation of the DNA repair enzyme Gadd45 (Grace et al. 2007), quiescence of the cell cycle at the G0/G1 phase (Tamulevicius et al. 2007), and inflammation suppression (Comalada et al. 2006; Ha et al. 2013). In addition, clinical data indicate that genistein can reduce intestinal, urinary, and sexual adverse effects when used in combination with radiation therapy to treat prostate cancer (Ahmad et al. 2010).

### Hesperidin

Hesperidin (hesperetin-7-rhamnoglucoside) is a flavanone glycoside belonging to the flavonoid family. Hesperidin is the major flavonoid in lemons and sweet oranges and has radioprotective effects mediated by its anti-inflammatory and antioxidant properties. Specifically, it can prevent oxidative stress damage caused by IR exposure to lung tissue. Additionally, hesperidin was shown to protect against genetic damage to lymphocytes induced by the radiotracer 99mTc-MIBI in vitro (Fardid et al. 2016). In addition, hesperidin inhibited IR responses in rats (Rezaeyan et al. 2016) and demonstrated antioxidant and anti-apoptotic activities in mouse testes injured by IR (Shaban et al. 2017).

### Lycopene

Lycopene is a widely distributed dietary carotenoid found in red fruits and vegetables, such as tomatoes, pink guava, pink grape fruit, apricots, watermelon, and papaya (Gupta et al. 2015), and has demonstrated potential protective effects against IR damage. The antioxidant activities of lycopene have been extensively evaluated in vitro and in vivo and are mediated by its ability to scavenge ROS (Srinivasan et al. 2009). Previous studies also suggest that lycopene could reduce IR damage by removing singlet oxygen and scavenging free radicals (Kelkel et al. 2011). Lycopene pretreatment significantly decreased the frequency of micronuclei, dicentric aberration, and translocation in irradiated human lymphocytes and rat hepatocytes. IR-induced lipid peroxidation was also decreased by lycopene pretreatment, and the activity of antioxidant enzymes including SOD, catalase, and glutathione peroxidase was increased (Gajowik and Dobrzynska 2017; Srinivasan et al. 2009; Srinivasan et al. 2007).

### N-Acetyl tryptophan glucopyranoside (NATG)

N-acetyl tryptophan glucoside (NATG), a bacterial secondary metabolite, was purified from the radioresistant bacterium *Bacillus subtilis*. NATG pretreatment mediates radioprotection by preventing IR-induced apoptosis through increase of the cytoprotective cytokines IFN-γ, IL-17A, and IL-12 (Malhotra et al. 2015). NATG pretreatment has also been demonstrated to protect J774A.1 macrophages against IR-induced DNA damage (Malhotra et al. 2016) and was able to significantly enhance antioxidant enzymes in response to IR-induced damage in murine macrophages (Malhotra et al. 2018).

### Sesamol

Sesamol (3,4-methylenedioxyphenol), a component of sesame seeds and sesame oil, is a natural phenolic antioxidant (Joshi et al. 2005). Sesamol possesses strong ROS scavenging and antioxidant properties (Kanimozhi and Prasad 2009) and is able to protect against IR-induced DNA damage in human lymphocyte cells (Prasad et al. 2005). Additionally, sesamol significantly attenuates IR-induced DNA damage in the hematopoietic system of mice (Kumar et al. 2018), and reduces genotoxicity in bone marrow cells (Kumar et al. 2015). Further, it protects the gastrointestinal and hematopoietic systems against IR-induced injury in mice (Khan et al. 2015). The radioprotective effects of sesamol are mediated by ROS scavenging (Mishra et al. 2011) and enhancement of DNA repair activity (Nair and Nair 2010).

### Psoralidin

Psoralidin is a natural phenolic compound that can be isolated from *Psoralea corylifolia,* which is widely used in traditional medicine. Structurally, psoralidin contains two hydroxyl groups, one conjugated lactone, one isopentenyl group, and an intramolecular ether linkage. Psoralidin exhibits a variety of biological activities including anti-inflammatory (Yang et al. 2011), antioxidant (Pahari and Rohr 2009), and antitumor (Bronikowska et al. 2012) activities. Psoralidin also regulates the NF-κB pathway and PI3K-mediated Akt signaling pathway (Kumar et al. 2009). Additionally, psoralidin inhibits Syk-mediated activation of the PI3K-IKK-IκB signaling pathway (Chiou et al. 2011). Psoralidin has demonstrated potential as a radioprotector due to its anti-inflammatory effects in human lung fibroblasts and mice. Specifically, psoralidin decreased IR-induced cyclooxygenase (COX2) expression through regulation of the NF-κB and PI3K/Akt pathways. Psoralidin also decreased IR-induced pro-inflammatory cytokines such as TNF-α, TGF-β, IL-6, IL-1 α/β, and ICAM-1 (Yang et al. 2011).

### Troxerutin

Troxerutin is one derivative of the flavonoid rutin, which is isolated from *Sophora japonica* (Japanese pogoda tree), and it has been used for the treatment of chronic venous insufficiency disease (Adam et al. 2005; Vanscheidt et al. 2002). Troxerutin has been shown to improve capillary function and to reduce capillary fragility and abnormal leakage. Moreover, troxerutin has demonstrated anti-thrombotic, anti-erythrocytic, and fibrinolytic activity (Maurya et al. 2005). Studies indicate that troxerutin is safe and effective in elderly patients (Marhic 1991) and pregnant women (Wijayanegara et al. 1992). Troxerutin scavenges oxygen-derived free radicals (Kessler et al. 2002; Wenisch and Biffignandi 2001). During head and neck cancer radiotherapy, administration of a mixture of coumarin and troxerutin conferred protection to the oral mucosa and salivary glands (Grotz et al. 2001). Moreover, troxerutin has been shown to inhibit lipid peroxidation in the membranes of subcellular organelles and in normal tissue of tumor-bearing mice exposed to IR. Furthermore, administration of troxerutin resulted in protection of DNA in whole body-irradiated tumor-bearing mice. Importantly, troxerutin protected DNA in blood leukocytes and bone marrow cells, but it did not protect DNA in tumor cells. Troxerutin also enhanced DNA repair and conferred dose-dependent radioprotection to mouse blood and BM cells (Maurya et al. 2004), and it inhibited micronuclei in mouse blood reticulocytes and human peripheral blood lymphocytes. Suggested molecular mechanisms of radioprotection by troxerutin include activation of AKT and inhibition of JNK, which result in reduced IR-induced PTEN activation (Xu et al. 2017).

### Vanillin

Vanillin (4-hydroxy-3-methoxybenzaldehyde), the major component of natural vanilla, is one of the most widely used flavoring materials worldwide. The source of vanilla is the pod, or bean, of the tropical Vanilla orchid (*Vanilla planifolia* Andrews, *V. fragrans* Salisb. Ames). Vanillin has demonstrated antioxidant activities including inhibition of lipid peroxidation and scavenging of hydroxyl radicals (Harish et al. 2005). Vanillin and its analogs have also demonstrated anti-mutagenic or anti-genotoxic effects in cells and animals (King et al. 2007; Shaughnessy et al. 2001). Interestingly, oral administration of vanillin to mice after injection of mitomycin C decreased the frequency of micronucleated polychromatic erythrocytes by 50% (Inouye et al. 1988). Data suggest that the anti-mutagenic effects of vanillin may result from a mutation-dependent, error-free pathway for post-replication DNA repair (Ohta et al. 1988). This pathway inhibited chromosomal aberrations induced by IR in mice (Sasaki et al. 1990) and in V79 cells (Keshava et al. 1998), inhibited lipid peroxidation in rat liver mitochondria, and reduced DNA damage in the plasmid pBR322 (Kumar et al. 2000). Additionally, the vanillin derivative VND3207 (4-hydroxy-3,5-dimethoxybenzaldehyde) reduced IR damage through antioxidant activities. Specifically, VND3207 treatment before IR significantly reduced DNA damage and activated the Akt pathway to induce survival (Zheng et al. 2008).

### Zingerone

Zingerone is present at a level of about 9% in ginger (*Zingiber officinale*), which is the most commonly used spice worldwide. Zingerone demonstrates potent pharmacological activities including antioxidant, anti-inflammatory, anticancer, and antimicrobial activities (Ahmad et al. 2015). Zingerone treatment to human lymphocytes prior to IR reduced micronuclei, apoptosis, and ROS generation, and alkaline comet tail determination also showed decreased comet tail moments (Rao et al. 2011). Further, zingerone also protected against IR-induced oxidative stress and DNA damage in Chinese hamster fibroblast cells (Rao and Rao 2010), demonstrating its potential as a possible radioprotector.

### Zymosan A

Zymosan A is a glucan derived from the cell wall of *Saccharomyces cerevisiae*. Zymosan contains repeating glucose units connected by β-1,3-glycosidic linkages. In macrophages, zymosan A induced pro-inflammatory cytokines, arachidonate mobilization, inositol phosphate formation, and protein phosphorylation (Sato et al. 2003). Zymosan A exhibits radioprotective effects through regulating the immune system and inflammatory response. Specifically, zymosan A activates Toll-like receptor 2 on macrophages to induce inflammatory signaling. Toll-like receptor 2 has a critical role in recognizing specific components of pathogenic microorganisms (Underhill 2003) and cooperates with Toll-like receptor 6 and CD14 in response to zymosan A. Zymosan A is also recognized by Dectin-1, and the recognition of zymosan A triggers macrophages and dendritic cells to induce inflammatory signals (Gantner et al. 2003; Ozinsky et al. 2000). Zymosan A protected mouse cells from radiation-induced apoptosis by upregulating cytokines such as G-CSF, GM-CSF, IL-6, and IL-12. Zymosan A also protected cells from IR-induced DNA damage and reduced the number of γ-H2AX foci that were caused by IR (Du et al. 2018).

## Conclusion

An ideal radioprotector should be easily available, affordably priced, and not result in serious toxicities over a wide dose range. It should also demonstrate an absence of cumulative effects from repeated treatments, be able to be orally administered, have a protective effect on widespread organ systems, and demonstrate efficacy for different types of radiation (X, gamma, electron, and neutron). Finally, it should possess a reasonable dose reduction factor and an ability to act through multiple mechanisms (Cheki et al. 2016). A large number of pharmacological agents are currently being developed to prevent, mitigate, or treat IR-induced toxicities. Even though the use of radioprotectors is a very promising approach for both accidental and therapeutic exposure, no available radioprotectors are capable of completely preventing IR-related toxicities. Therefore, the use of naturally occurring compounds may be a good strategy in the development of ideal radioprotectors.

The work summarized here proposes that multiple potential radioprotectors exist with different targets and mechanisms. Radioprotectors mitigate IR-induced injury and/or IR-related syndromes by preventing or destroying free radicals, activating enzymes involved in the repair of DNA breaks, stimulating hematopoiesis and the immune system, or interacting with proteins in signaling and apoptotic execution pathways (Fig. 5; Table 2). These radioprotective agents include both synthetic chemical compounds and natural products. However, naturally occurring compounds have notable benefits for use in radioprotection. The major advantage of using naturally occurring compounds is their increased safety relative to synthetic chemical compounds. Moreover, natural products have been shown to be effective in curing symptoms similar to those of radiation syndrome. Studies exploring these compounds as a novel approach to radioprotection have demonstrated the effectiveness of plant-derived compounds in treating various human ailments all over the world.

Considering the pressing need for efficient and safe medicinal resources and the broad range of circumstances in which radioprotectors are required, future efforts to develop natural radioprotectors remain extremely important.
