# Correction to: Pharmacology of natural radioprotectors

**DOI:** 10.1007/s12272-019-01194-1

**Published:** 2019-12-12

**Authors:** Gil-Im Mun, Seoyoung Kim, Eun Choi, Cha Soon Kim, Yun-Sil Lee

**Affiliations:** 1 Graduate School of Pharmaceutical Sciences, Ewha Womans University, Seoul, 120-750 Korea; 2 Radiation Health Institute, Korea Hydro & Nuclear Power CO., LTD, 172, Dolmr-ro, Seongnam-si, Gyeonggi-do 13605 Korea

## Correction to: Arch. Pharm. Res. 10.1007/s12272-018-1083-6

We apologize that there are some errors in the references for three sentences and table 2.

1. In the section “Naturally occurring radioprotectors”, the reference of the first paragraph’s last sentence should be changed from ‘(Yu et al. 2003)’ to ‘(Praetorius and Mandal 2008)’.

Indeed, amifostine has several clinically relevant limitations, including (1) an administration time within a narrow window (15–30 min before IR exposure); (2) approval only for intravenous (IV) administration (Praetorius and Mandal 2008); and (3) high toxicity associated with undesirable side effects including nausea, vomiting, cephalalgia, and hypotension.

2. In the section “Ferulic acid”, the reference of sixth sentence should be changed from ‘(Srinivasan et al. 2006)’ to ‘(Prasad et al. 2006)’.

Pretreatment of lymphocytes and hepatocytes with ferulic acid resulted in a significant decrease in DNA damage and lipid peroxidation after IR exposure (Prasad et al. 2006).

3. In the section “Hesperidin”, the reference of fourth sentence should be changed from ‘(Fardid et al. 2016)’ to ‘(Hosseinimehr et al. 2009)’.

Additionally, hesperidin was shown to protect against genetic damage to lymphocytes induced by the radiotracer 99mTc-MIBI in vitro (Hosseinimehr et al. 2009).

4. In connection to these errors, the following articles should be deleted in the References section:
  Fardid R, Ghorbani Z, Haddadi G, Behzad-Behbahani A, Arabsolghar R, Kazemi E, Okhovat MA, Hosseinimehr SJ (2016) Effects of hesperidin as a radio-protector on apoptosis in rat peripheral blood lymphocytes after gamma radiation. J Biomed Phys Eng 6:217–228
  Srinivasan M, Rajendra Prasad N, Menon VP (2006) Protective effect of curcumin on gamma-radiation induced DNA damage and lipid peroxidation in cultured human lymphocytes. Mutat Res 611:96–103
  Yu Z, Eaton JW, Persson HL (2003) The radioprotective agent, amifostine, suppresses the reactivity of intralysosomal iron. Redox Rep 8:347–355

5. Instead, following articles should be added to the References section:
  Hosseinimehr SJ, Ahmadi A, Beiki D, Habibi E, Mahmoudzadeh A (2009) Protective effects of hesperidin against genotoxicity induced by 99mTc-MIBI in human cultured lymphocyte cells. Nucl Med Biol 36:863–867
  Praetorius NP, Mandal TK (2008) Alternate delivery route for amifostine as a radio-/chemo-protecting agent. J Pharm Pharmacol 60:809–815
  Prasad NR, Srinivasan M, Pugalendi KV, Menon VP (2006) Protective effect of ferulic acid on gamma-radiation-induced micronuclei, dicentric aberration and lipid peroxidation in human lymphocytes. Mutat Res 603:129–134

6. In Table 2, three references should be corrected.
  ‘Hall and Giaccia (2012)’ for caffeine should be corrected to ‘Kamat et al. (2000)’.
  ‘Farooqi and Kesavan (1992)’ should be added for caffeine.
  ‘Fardid et al. (2016)’ for hesperidin should be corrected to ‘Shaban et al. (2017)’.

**Table 2:** List of naturally occurring compounds with radioprotective effects

Natural compounds	Source	Radioprotective effects	References
Apigenin	Parsley, Celery, Chamomile	Anti-inflammatory, anti-proliferative, and anti-progression	Begum et al. (2012)
Bergenin	*Caesalpinia digyna*	Activation of the MAP kinase and ERK pathways and protection against radiation damage	Dwivedi et al. (2017) Veerapur et al. (2009)
Caffeine	Coffee beans	Protection against the oxic component of damage in rat liver mitochondria Inhibition of radiation-mediated chromosomal aberrations in mouse bone marrow cells	Farooqi and Kesavan (1992) Kamat et al. (2000)
Chlorogenic acid/quinic acid	Cinchona bark, Coffee beans	Anti-inflammation, anti-mutagenic, DNA damage inhibition, and anti-oxidation	Cinkilic et al. (2013)
Coniferyl aldehyde	*Eucommia ulmoides*	Induction of heat shock transcription factor 1 and protection against radiation damage	Kim et al. (2015) Nam et al. (2013)
Curcumin	Turmeric root	Reduction of gastrointestinal symptoms during chemotherapy and radiation therapy Reduction of mucositis during radiation therapy Reduction of radiation dermatitis and desquamation	Verma (2016)
Delphinidin	Carrot, Tomato, Red onion, Cranberries, Concord grapes, etc	Anti-oxidation and anti-inflammation	Jeong et al. (2016) Watson and Schonlau (2015)
Epigallocatechin-3-gallate	*Camellia sinensis*	Increased levels of several anti-oxidant enzymes Protection of skin cells against radiation-induced damage and radioprotective effects against several radiation-mediated responses	Zhang et al. (2016) Zhu et al. (2016)
Ferulic acid	Rice, Green tea, Coffee beans	Protection against radiation-induced damage and enhancement of DNA repair Prevention of radiation-induced micronuclei and dicentric aberrations in human lymphocytes Enhancement of survival in mice after radiation	Das et al. (2014) Kikuzaki et al. (2002) Zhao et al. (2003)
Genistein	*Genista tictoria*, etc.	Protection against acute radiation injury Multiple mechanisms (e.g., antioxidant, free radical scavenger, anti-inflammatory, activation of the DNA repair enzyme Gadd45	Ahmad et al. (2010) Davis et al. (2007) Grace et al. (2007)
Hesperidin	Citrus fruit	Efficient radioprotection in rat lung tissue Protection of lipid peroxidation during radiation-induced tissue damage in rats	Rezaeyan et al. (2016) Shaban et al. (2017)
Lycopene	Tomato, Watermelon, Pink grapefruit, Papaya, etc	Protection against radiation-induced chromosomal damage in human lymphocytes Increased survival after radiation exposure	Kelkel et al. (2011) Srinivasan et al. (2009)
N-Acetyl tryptophan glucopyranoside (NATG)	*Bacillus* sp. INM-1	Overcoming radiation-induced apoptosis by improving cytoprotective cytokines Enhancement of antioxidant enzymes against radiation-induced damage	Malhotra et al. (2015) Malhotra et al. (2018)
Psoralidin	*Psoralea corylifolia*	Inhibition of radiation-induced PI3K-IKK-IκB signaling pathway and COX-2 expression Suppression of radiation-induced expression of pro-inflammatory cytokines	Chiou et al. (2011) Yang et al. (2011)
Sesamol	Sesame seeds, Sesame oil	Strong ROS scavenging and antioxidant properties	Kanimozhi and Prasad (2009) Mishra et al. (2011)
Troxerutin	*Sophora japonica*	Protection against radiation-induced damage to the salivary glands and mucosa Inhibition of lipid peroxidation in the membranes of subcellular organelles Differential protection of normal cells in irradiated tumor-bearing mice: protection in blood leukocytes and bone marrow cells but not in tumor cells	Maurya et al. (2004)
Vanillin	*Vanilla orchid* (*Vanilla planifolia, V. fragrans*)	Suppression of radiation-induced chromosomal aberrations in cells and in mice Anti-mutagenic effects	Kumar et al. (2000)
Zingerone	Ginger	Anti-oxidation	Ahmad et al. (2015) Rao and Rao (2010)
Zymosan A	*Saccharomyces cerevisiae*	Protection from radiation-induced apoptosis by upregulating the levels of cytokines Protection of cells from radiation-induced DNA damage	Du et al. (2018)
7. In the section “N-Acetyl Tryptophan Glucopyranoside (NATG)”, the word of first sentence should be changed from ‘*Bacillus subtilis*’ to ‘*Bacillus* sp. INM-1’.

